# Role of Nuclear Imaging to Understand the Neural Substrates of Brain Disorders in Laboratory Animals: Current Status and Future Prospects

**DOI:** 10.3389/fnbeh.2020.596509

**Published:** 2020-12-11

**Authors:** Annunziata D'Elia, Sara Schiavi, Andrea Soluri, Roberto Massari, Alessandro Soluri, Viviana Trezza

**Affiliations:** ^1^Institute of Biochemistry and Cell Biology, National Research Council of Italy (CNR), Rome, Italy; ^2^Section of Biomedical Sciences and Technologies, Department of Science, University “Roma Tre”, Rome, Italy

**Keywords:** small animal imaging, behavioral neuroscience, SPECT, PET - positron emission tomography, neuroimaging, brain disorders

## Abstract

Molecular imaging, which allows the real-time visualization, characterization and measurement of biological processes, is becoming increasingly used in neuroscience research. Scintigraphy techniques such as single photon emission computed tomography (SPECT) and positron emission tomography (PET) provide qualitative and quantitative measurement of brain activity in both physiological and pathological states. Laboratory animals, and rodents in particular, are essential in neuroscience research, providing plenty of models of brain disorders. The development of innovative high-resolution small animal imaging systems together with their radiotracers pave the way to the study of brain functioning and neurotransmitter release during behavioral tasks in rodents. The assessment of local changes in the release of neurotransmitters associated with the performance of a given behavioral task is a turning point for the development of new potential drugs for psychiatric and neurological disorders. This review addresses the role of SPECT and PET small animal imaging systems for a better understanding of brain functioning in health and disease states. Brain imaging in rodent models faces a series of challenges since it acts within the boundaries of current imaging in terms of sensitivity and spatial resolution. Several topics are discussed, including technical considerations regarding the strengths and weaknesses of both technologies. Moreover, the application of some of the radioligands developed for small animal nuclear imaging studies is discussed. Then, we examine the changes in metabolic and neurotransmitter activity in various brain areas during task-induced neural activation with special regard to the imaging of opioid, dopaminergic and cannabinoid receptors. Finally, we discuss the current status providing future perspectives on the most innovative imaging techniques in small laboratory animals. The challenges and solutions discussed here might be useful to better understand brain functioning allowing the translation of preclinical results into clinical applications.

## Introduction

In recent times, imaging technologies have been increasingly applied to laboratory animals in order to study biological processes in real time and at the molecular level. The term *Small Animal Imaging* is mainly referred to systems for imaging in mice and rats. Molecular imaging is a research area which, as defined by Weissleder and Mahmood (Weissleder and Mahmood, 2001), aims to characterize and measure *in vivo* biological processes at cellular and molecular level in laboratory animals. In fact, molecular imaging, in contrast with classical imaging diagnostics, focuses on investigating the functional anomalies that underlie diseases rather than evaluating the final effects of these alterations (Grassi et al., 2009).

To date, several rodent models of human diseases exist, with face, construct and predictive validity for the corresponding human condition (Willner, 1984; Van Der Staay et al., 2009; Blanchard et al., 2013; Stewart and Kalueff, 2015).

In neuroscience research, animal models are excellent translational research tools since they allow: (1) To dissect the role of genetic and environmental factors in the pathogenesis of brain disorders; (2) To unravel the relationships between altered brain function and behavior; (3) To validate new therapeutic targets and treatments. In parallel with the development of animal models of human diseases, innovative *in vivo* imaging technologies have been exploited to investigate the cellular and molecular mechanisms underlying such diseases.

The aim of this review is to investigate the role of SPECT/PET small animal imaging systems in order to understand the neural underpinnings of brain disorders. Several topics are discussed highlighting the strengths and weakness of both technologies. The application of some of the radioligands developed for small animal nuclear imaging studies is mentioned. As an example of the use of neuroimaging techniques in rodents to study neurotransmitter activity in various brain areas during task-induced neuronal activation, we focus on the imaging of opioid, dopaminergic, and cannabinoid receptors, as these neurotransmitter systems are highly involved in complex behaviors and their role in brain diseases has been extensively studied in rodents (Grace, 2016; Nummenmaa and Tuominen, 2018; Cristino et al., 2020).

Finally, potential developments in the field will be discussed, providing future perspectives on the most innovative imaging techniques in small laboratory animals.

## Molecular Imaging Techniques

**Figure 1:**
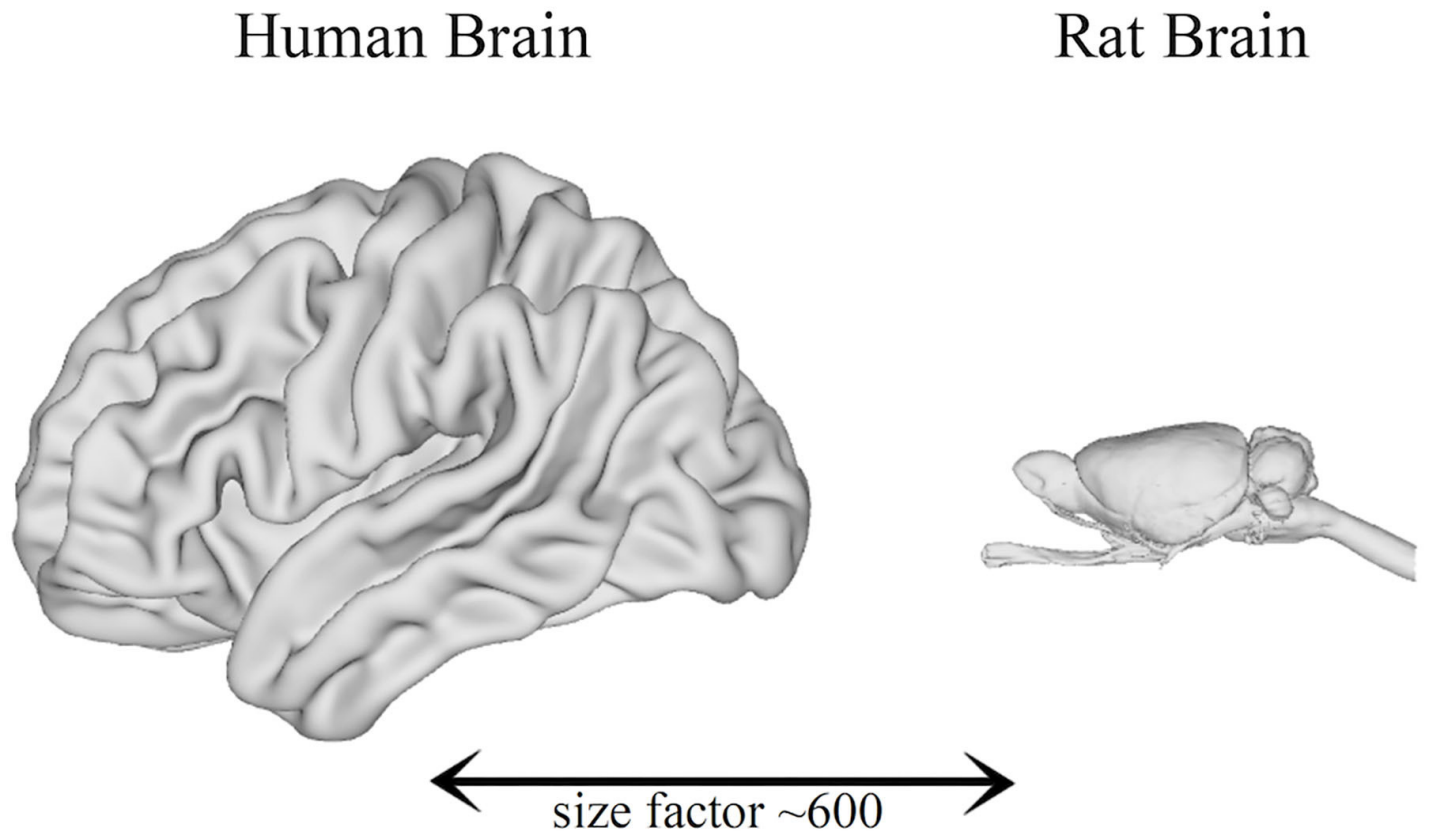
Comparison between the brain size of humans and rats (Bakker et al., 2015).

Significant progress in non-invasive imaging technologies has been made in the last decade. Some research groups began to develop systems with the aim of visualizing anatomical structures that are much smaller than those of man. The devices used in clinical practice are, in fact, inadequate given the small size of the organs of laboratory animals: indeed, the human brain has a volume of about 1,200 cm^3^, while the rat and mouse brains have, respectively, volumes of about 2 and 0.5 cm^3^, meaning that their brains are ~600 and 2400 times smaller than the human brain (Herfert et al., 2020) (Figure 1).

Because of the size of the animals involved, imaging devices for laboratory animals need to meet specific spatial resolution and sensitivity requirements and the signal must be optimized comparing with humans. Consequently, if imaging studies in laboratory animals are to be equivalent to human studies in terms of quality, the spatial resolution of the animal studies should be about 10 times better. In this prospective, small animal imaging acts as a non-invasive method to discern biological structures and functions *in vivo*, thus providing quantitative data in both healthy animals and in models of human diseases. Due to its non-invasive nature, a crucial aspect of molecular imaging is represented by its ability to systematically test rodent models of human diseases over the entire natural history of the disease under investigation, from inception to progression, and monitoring the effectiveness of pharmacological treatment or other interventions. Moreover, the overall number of the experimental animals required for a particular study can also be drastically reduced as each animal is able to function as its own control with its own biological variability.

### Small Animal Imaging Scanners: An Overview

Over the last decades, small animal imaging has become an integral part of molecular medicine. In fact, the study, diagnosis, and treatment of pathologies aided by the use of nuclear imaging have availed of significant degree of innovation, starting with the development of Computed Tomography (CT), Positron Emission Tomography (PET) and Single Photon Emission Computed Tomography (SPECT). The images produced by the last two techniques express the biodistribution of specific radioactive tracers (contrast agents) administered to the organism under study, and, unlike the CT scan (obtained through irradiation with external X-rays), allow to investigate metabolic processes (functional) rather than presenting morphological aspects of the organ or sector investigated. Additionally, PET and SPECT systems are able to process *in vivo* imaging on a molecular scale (hence molecular imaging). These characteristics have acquired increasing importance in modern molecular medicine.

Typically, the imaging technologies have been divided into two main categories: *morphological* and *functional*.

*Morphological modalities*—which primarily depict the anatomical districts with excellent spatial resolution—include X-Rays, Magnetic Resonance Imaging (MRI), Computed Tomography (CT), and Ultrasound (US).

*Functional modalities*—whose information specifically regards metabolism and biochemistry—include Magnetic resonance spectroscopy imaging (MRSI) and functional magnetic resonance imaging (fMRI), SPECT, PET and, optical imaging (bioluminescence and fluorescence).

In this context, PET and SPECT have given, over the last few years, a significant contribution to evaluate the physiological functions and biochemical changes of molecular targets. Both techniques are based on the measurement of a radionuclide decay, through which a positron or a γ-ray is emitted and thus generates photons. Furthermore, the advantages of PET and SPECT systems also lie in high sensitivity, good spatial resolution and limitless penetration depth, leading to their vital role in molecular imaging for both preclinical and clinical studies (Lu and Yuan, 2015).

### Advances in Imaging Systems

To date, new imaging tools and applications in small laboratory animals are being developed. The use of these tools in preclinical research is crucial to identify and develop new diagnostic or therapeutic drugs and facilitate the translation of preclinical findings to the clinics. Moreover, imaging of living animals enables longitudinal studies, allowing continuous, dynamic and sometimes nearly instantaneous identification/quantification of disease progression and, consequently, of treatment efficacy. In this way, the disease progression or the drug pharmacological effect can be monitored much more effectively. In addition, by using animals as their own controls, the imaging modalities reduce the number of animals sacrificed. This approach provides high statistical power by making use of a low number of animals, which cuts costs simultaneously respecting the ethical guidelines for the use of laboratory animals in research. Similarly, drugs at a very early stage of development or with limited effectiveness can be successfully identified with this type of imaging (Mejia et al., 2016).

In current research, a whole CT or MRI with high spatial resolution needs only seconds or few minutes to be completed. The application and the accumulation of contrast agents are intended to dynamically monitor tissue vascularisation, perfusion and permeability, thus enabling to collect functional data. By contrast, PET and SPECT imaging modalities, which are highly sensitive to administered radiolabelled molecules, allow the identification of metabolic and physiological activity of the tissues (Koba et al., 2013). Among the novel promising tools in constant development, molecular ultrasound, high field MRI as well as photoacoustic and optical imaging are playing a significant role (Fine et al., 2014).

Each of these imaging techniques presents a number of weaknesses and strengths. On the one side, MRI and CT have higher spatial resolution than SPECT and PET. On the other side, SPECT and PET have a better sensitivity with respect to structural modalities and can detect lower tracer concentration (picomolar or nanomolar range). However, both modalities are used to detect physiological or altered biochemical processes.

Radionuclide imaging is defined by two key elements, namely a bio-marker and an imaging device. The first element should have high specific and sensitive characteristics in order to study a molecular or cellular phenomenon. The second element, instead, is a radiation detector with the aim to localize activity distribution within the human or the animal body. Specifically, the imaging devices enable the investigation of molecular, metabolic and functional parameters due to the variety of available specific radiopharmaceuticals (Mannheim et al., 2018). In this scenario, both PET and SPECT provide unique functional information at the molecular level. However, this sole information lacks proper correlation with anatomical structures. To this purpose, hybrid or fusion imaging modalities, such as PET/MRI, PET/CT, SPECT/CT and SPECT/MRI, are able to combine the functional and morphological details. As a result, the flexibility and high chemical sensitivity of SPECT/PET perfectly suits the extraordinary soft tissue contrast and high resolution of MRI/CT (Kagadis et al., 2010).

## SPECT

The SPECT technique is based on the detection of single- gamma photon emissions (Wirrwar et al., 2001; Peremans et al., 2005; Aoi et al., 2006; Chen et al., 2017). The conventional Gamma Camera – invented by Hal Anger in 1957 – stands out as the core instrument in SPECT imaging (Hantraye, 1998). Originally the gamma camera was intended as a detection technique to localize an activity distribution of an administered radionuclide. Basically, it is a gamma ray position sensitive detector that typically consists of a scintillator crystal (a single monolithic element or a pixelated matrix) optically coupled to an array of photodetectors, read by a position and energy determination circuitry. The spatial correlation between detected events and gamma ray emission site is provided by a multi-hole collimator mounted on the front face of the system (Khalil et al., 2011).

The gamma camera detects the incoming γ-rays by their interactions with the scintillation crystal, which is usually a NaI(Tl) (thallium-doped sodium iodide) crystal. The energy absorbed by the scintillation crystal in the photon interactions is converted to measurable flashes of light. The amount of scintillation light is then converted in electrical signals by an array of sensitive photodetectors, such as photomultiplier tubes (PMTs). The relative response in each PMT to each event allows to produce two signals (X and Y) which give the position of the scintillation, and one signal (Z) that represents the energy deposited in the crystal. Finally, the electrical signals are processed by an imaging computer.

The scintillation crystal element is paired with the collimator, which is used to select the direction of the detected γ-rays. The most used collimator consists of a heavy metal (e.g., lead or tungsten) plate containing a large number of holes whose orientation can be parallel, diverging or converging toward the camera face depending on the specific collimator design. Only photons traveling along a hole axis will interact with the crystal, while γ-rays not traveling in the proper direction are absorbed by the collimator.

Another type of collimator that is frequently used is the pinhole collimator. It simply consists of a small hole (typically a few millimeters) in a piece of heavy metal absorber. Only γ-rays passing through the hole project an image of the source onto the scintillation crystal. The projected image is magnified when the distance of the source to the hole is smaller than that of the hole from the crystal, and minified in the opposite case.

**Figure 2:**
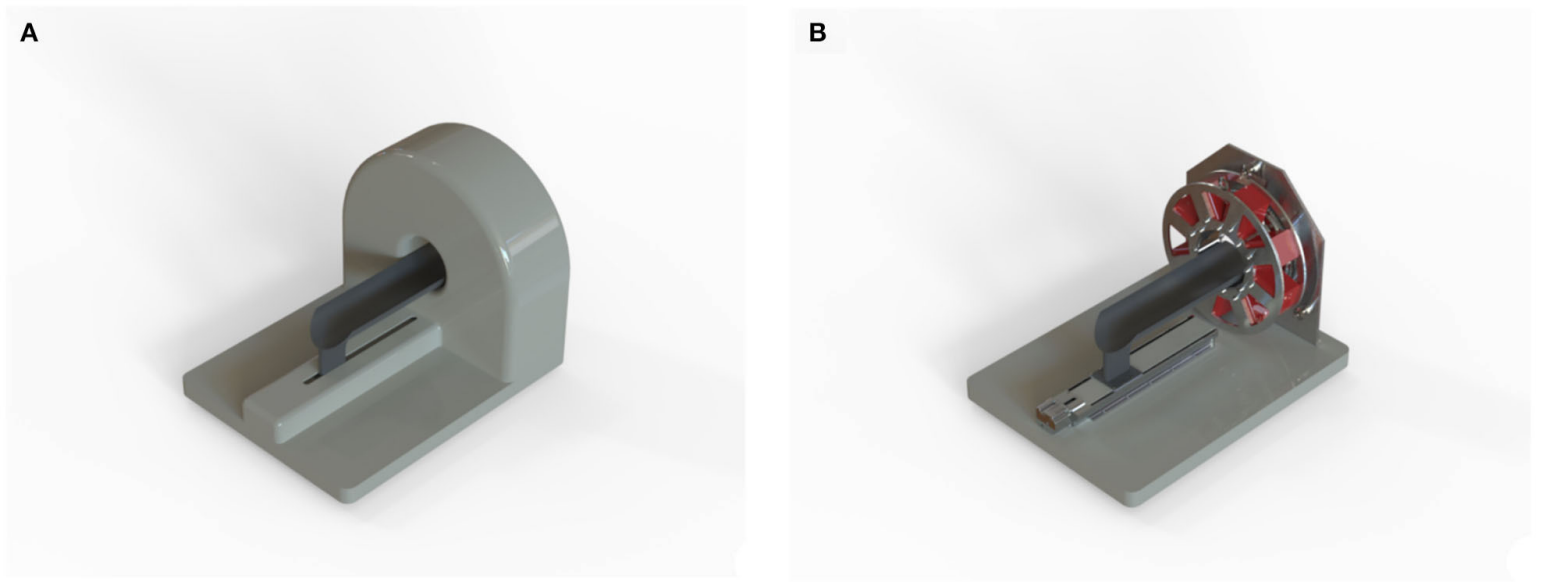
Dedicated SPECT system for *in-vivo* preclinical molecular imaging; **(A)** 3D rendering of the system. **(B)** Inner structure that shows the eight detector heads mounted on a rotary stage.

Whereas, Planar Scintigraphy consists of a planar acquisition of the 2D projection of a tracer distribution, SPECT imaging produces 3D tomographic images generated by the rotation of a gamma camera around an object. Then, by using specific reconstruction algorithms, the resulting projections at different angles are merged into a 3D image (Jaszczak and Coleman, 1985; Rogers and Ackermann, 1992; Spanoudaki and Ziegler, 2008; Peterson and Shokouhi, 2012; Lauber et al., 2017). In Figure 2 is shown the design of a preclinical SPECT system.

Usually, preclinical SPECT systems apply pinhole collimation to obtain better spatial resolution by pinhole magnification. Moreover, to increase sensitivity most systems employ multiple pinholes (Peterson and Furenlid, 2011). In order to increase resolution and sensitivity, also different crystal materials such as CsI(Na) or LaBr3(Ce) have been implemented (Meikle et al., 2005). Modern systems are able to detect minute tracer concentrations (0.1 nanomol) with submillimeter resolution (0.5–0.7 mm) *in vivo* (Peterson and Furenlid, 2011; Peterson and Shokouhi, 2012). Some preclinical SPECT systems also incorporate semiconductor detector materials (CdTe or CdZnTe [CZT]) offering the possibility of higher energy discrimination efficiency, which are important for low-energy radionuclides (e.g. ^125^I) and dual isotope applications (Lauber et al., 2017).

In the last decades, small animal SPECT performance has been significantly improved, taking into account spatial resolution. However, a number of challenges, such as increasing the detection efficiency without sacrificing spatial resolution, are still open.

SPECT has been playing an increasingly relevant role in the molecular imaging matrix thanks to its ability to detect biological processes *in vivo*. Furthermore, it boasts the capability to imaging two or more radio-labeled molecules simultaneously which may be particularly useful when studying complex molecular interactions and the sequencing of cellular events (Meikle et al., 2005). Despite notable progress in recent developments in small animal SPECT imaging, new innovation challenges involve the field of camera sensitivity, spatial resolution, and image reconstruction and quantification.

Several solutions were exploited before the pinhole design became widely used in small animal imaging SPECT systems, such as the YAP-(S)PET (Del Guerra et al., 1998) and the TierSPECT (Schramm et al., 2000). Furthermore, a common choice was to pair a pinhole collimator with a single scintillation camera (Habraken et al., 2001).

Usually, a high radiation dose level was applied to the animal (Vastenhouw and Beekman, 2007) because of the long acquisition times required with moderately elevated levels of radioactivity (e.g., 37 MBq [1 mCi] of^99*m*^Tc).

Small-animal SPECT, still for the most part, depends on pinhole collimation to accomplish millimeter or submillimetre spatial resolution, nevertheless there is a compromise between high resolution and lower sensitivity when utilizing pinhole collimation secondary to the limited number of photons allowed through the pinhole to the face of the detector. The sensitivity of the single-pinhole system relies upon the pinhole size yet is ordinarily of about 1% or less for even larger pinholes (e.g., 3 mm). The use of multi-pinhole collimation in addition with multiple detectors have improved this compromise between spatial resolution and sensitivity, speeding up and upgrading volume detection for small γ-emitting sources injected in small volume (rodents). Be that as it may, the sensitivity of small animal SPECT may never arrive at the sensitivity of small animal PET (at present on the request for 1–10% for business imaging frameworks) (Vastenhouw and Beekman, 2007; Franc et al., 2008).

## PET

The PET technique is based on the coincident detection of the two annihilation photons originating from positron emitting radioisotopes (Fahey, 2002; Tai and Laforest, 2005; Torres Espallardo, 2017).

Similarly with SPECT tomography, positron emission tomography exploits the detection of γ-photons. In order to detect the positrons (subatomic e^+^ particles) emitted from the tracers, the two γ-photons coming from the annihilation between positrons and electrons of the matter are detected. The annihilation is the process undertaken by the collision of a positron and an electron: these particles are replaced by two γ-photons of fixed energy (511 keV), which travel along the same line but in opposite directions (at a 180° angle) (Kagadis et al., 2010). The kinetic energy of the emitted positron determinates the “positron range” i.e., the distance traveled before annihilation (few millimeters). For this reason, PET systems are usually characterized by a full ring of detectors in order to identify the opposite γ-photons generated by the annihilation. Considering that photons travel at the speed of light, the opposing detectors on the ring will receive a signal almost simultaneously (Turkington, 2001). Practically, the absence of a physical collimator implies that the detection is carried out within a short time window (usually 4–20 ns) which determines the trajectory of the annihilation point. This kind of electronic collimation improves the sensitivity of the system, which can be up to 100 times greater than SPECT, especially when 3D data are obtained (Kagadis et al., 2010). Nevertheless, unlike SPECT, it is physically impossible to improve spatial resolution above a certain threshold due to the methodical background of a positron range (Turkington, 2001). Thanks to the contribution of several advancements, such as replacing bismuth germanate (BGO) scintillators by improved detector blocks made of inorganic scintillators, for example lutetium oxyorthosilicate (LSO), lutetium–yttrium oxyorthosili-cate (LYSO) or gadolinium oxyorthosilicate (GSO), the latest micro-PET systems for preclinical studies have reached values of spatial resolution of about 1.0 mm under optimal conditions (Hutchins et al., 2008). Furthermore, PET signal acquisitions are also facilitated by the use of novel compact photodetectors, based on semiconductor technology, by further reducing the examination time and the signal to noise ratio (SNR) in a significant way (Levin and Zaidi, 2007; Pichler et al., 2008; Herzog and Lerche, 2016).

In Table 1 is illustrated a performance comparison between SPECT and PET systems.

**Table 1:** Performance comparison between SPECT and PET systems for preclinical imaging (adapted from Jansen and Vanderheyden, 2007).

**Advantages**	**Disadvantages**	**Radiation type**	**Spatial resolution^a^**	**Temporal resolution^b^**	**Sensitivity^c^**	**FOV^d^**	**Ability for brain imaging**	**Dual radionuclides**
**SPECT**								
Longer physical half-lives; multiple radionuclide detection, highest specific radioactivities, modular and more simple chemistry	Less sensitive than PET; most contrast agents are limited to large molecules; semiquantitative data only	Ionizing (γ^−^ radiation)	≤ 1 mm	Minutes	10^−10^-10^−11^	~ 8	Yes	Yes
**PET**								
High sensitivity; accurate quantification; diversity of available biological probes	Short-lived radionuclides; limited spatial resolution; dependence on local chemistry facilities (cyclotrons); expensive tracers and equipment	Ionizing (β^+^; γ^−^ radiation)	1–2 mm	Seconds - Minutes	10^−11^-10^−12^	~ 7	Yes	No

a *Spatial resolution expresses in millimeters, refers to the minimum distance that the imaging modality can differentiate two independently measured objects*;

b *Temporal resolution refers to the duration of time need to acquire enough events to form an image of a dynamic process*;

c *Sensitivity refers to the ability to distinguish a molecular probe from the background, the unit is mole per liter*;

d *FOV (Field of View) expresses in centimeters, refers to the dimensions of the exact anatomic region included in a scan*.

## Radiopharmaceuticals

The new field of molecular imaging is characterized by an increasing demand of innovative high-sensitive and specific imaging agents that can be rapidly translated from small animal models to humans. Radiopharmaceuticals are radioactive drugs; in particular, radiometals stand out as ionizing radiation sources which are able to diagnose or treat various diseases. Due to their wider range of nuclear properties (half-life, decay characteristics etc.), rich coordination chemistry and easy availability, this kind of radionuclides have acquired significant importance in comparison with other non-metallic (organic) radionuclides such as ^18^F, ^11^C, ^13^N, ^15^O, ^124^I. In this context, ^99m^Tc-radioimaging agents for myocardial perfusion, such as ^99m^Tc-sestamibi, boast great physic-chemical and nuclear properties (Bhattacharyya and Dixit, 2011). The most commonly used radionuclides used in SPECT/PET imaging are summarized in Table 2.

**Table 2:** Characteristics of the most commonly used radionuclides in SPECT/PET imaging.

	**Radionuclide**	**Half-life**	**Energy**	**Decay**
SPECT	^99m^TC	6.02 h	140 keV	IT (isomeric transition)
	^131^I	8.04 days	284, 364 keV	β^−^ (beta minus)
	^123^I	13.22 h	159 keV	EC (electron capture)
	^111^In	2.83 days	171, 247 keV	EC (electron capture)
	^201^Tl	3.05 days	68-80 keV	EC (electron capture)
PET	^18^F	110 min	511 keV	β^+^(beta plus)
	^11^C	20.3 min	511 keV	β^+^(beta plus)
	^68^Ga	68 min	511 keV	β^+^(beta plus)
	^13^N	10.0 min	511 keV	β^+^(beta plus)
	^15^O	2.07 min	511 keV	β^+^(beta plus)

Nuclear medicine research mainly utilizes radiopharmaceuticals that are aimed to assess the state of diseases or diseases themselves along with the effects of treatments. Within diagnostic imaging, SPECT and PET commonly make use of radiopharmaceuticals which gives them the ability to detect and serially monitor a variety of biological and pathophysiological processes, usually with tracer quantities of radiolabelled peptides, drugs, and other molecules at doses free of side effects (Blankenberg and Strauss, 2002, 2007; Khalil et al., 2011). Both techniques are well-established clinical imaging modalities providing high-quality sensitivity in deep tissue and are based on the injection of radiopharmaceuticals labeled with gamma emitting isotopes into the living subject.

**Figure 3:**
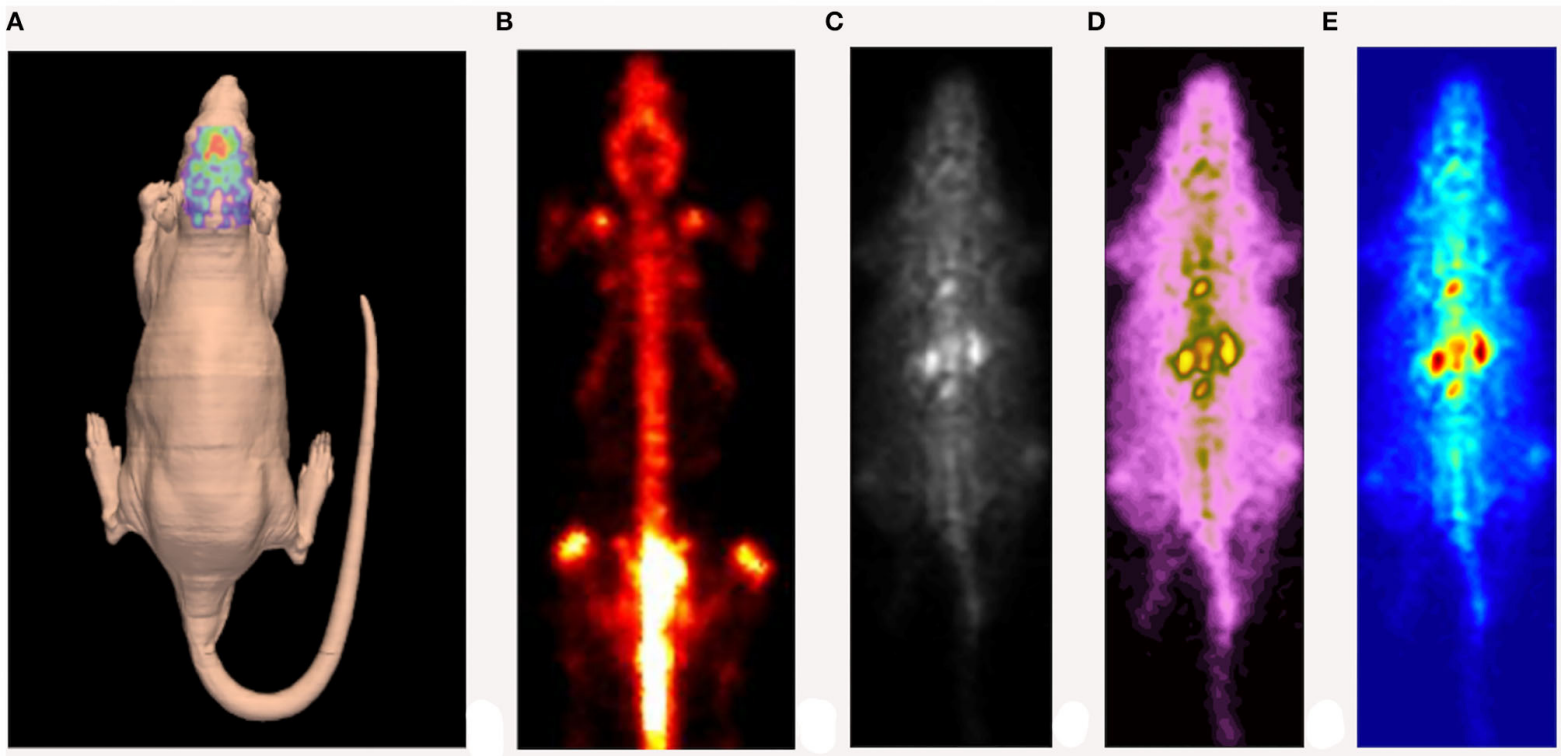
Example of high-resolution SPECT image of a rat injected with [^123^I] DATSCAN. **(A)** Reconstruction of the animal's skin carried out by a laser scanner. **(B)** SPECT image obtained 24 h after the injection. **(C–E)** Early SPECT image obtained ~1 h after the injection (different color maps).

Gamma-photon-emitting isotopes eligible for SPECT imaging are usually elements with higher atomic numbers [^99m^Tc (technetium), ^123^I (iodine), ^111^In (indium)], which are suitable for labeling several molecules: from simple organic chelates to proteins, antibodies, hormones or selectins. However, it may take a relatively long time for the diffusion and clearance of larger-molecule radiopharmaceuticals. To this purpose, radionuclides with a relatively longer half-life (hours) appear to be more appropriate (Meikle et al., 2005). Generally, SPECT is widely used in key fields of preclinical research, including cardiovascular research (Liu et al., 2002; Meoli et al., 2004; Tsui and Kraitchman, 2009), stem-cell research (Pomper et al., 2009; Rodriguez-Porcel, 2010), oncology (Hanahan and Weinberg, 2000, 2011; Ponsky et al., 2002), neuroscience research (Kung et al., 2004; Nikolaus et al., 2004, 2005b, 2009, 2011; Sharma and Ebadi, 2008) as well as for the development of new drugs (Rudin, 2009). An example of a high-resolution SPECT image is depicted in Figure 3.

The most frequent positron emitters used in PET are ^11^C (carbon), ^13^N (nitrogen), ^15^O (oxygen) and ^18^F (fluorine). These emitters share the big advantage of labeling almost any organic molecule without changing its chemical properties. However, the fact that these radiopharmaceuticals usually have a very short half-life (minutes) requires the need for producing and compounding radio-labels *in situ*, since the availability of dedicated facilities equipped with cyclotrons is bound to be limited. Among the PET emitters, ^18^F with its relatively longer half-life (110 min) allows external production and transportation (Koba et al., 2011, 2013; Fine et al., 2014). In recent times, ^124^I (half-life of 4.18 days) and other isotopes with longer half-life are being increasingly used in clinical diagnosis and post clinical studies (Kagadis et al., 2010). Despite these limits, PET is the most frequently used preclinical imaging modality for oncological research (Vavere and Lewis, 2007; Niu and Chen, 2009; Hoglund et al., 2011), cardiology (Tawakol et al., 2020), neurology (Schulz and Vaska, 2011; Schulz et al., 2011), drug development (Xi et al., 2011; Piel et al., 2014), as well as neuroscience and behavioral research (Jacobs et al., 2003; Schulz and Vaska, 2011; Vanitha, 2011; Zhu and Zhu, 2019). This is due to PET advantage of providing, along with higher sensitivity, the construction of physiological biomarkers labeled with positron emitting isotopes ^11^C, ^13^N, ^15^O, or ^18^F, in order for physiological and pathological processes to be monitored with no interference (De Kemp et al., 2010). Notwithstanding, the physical properties of e^+^ radiation (positron range, photon non-collinearity, and random events) limit the achievable spatial resolution (1–2.2 mm) (Lecomte, 2004; Tai et al., 2005). On the contrary, the achievable spatial resolution of modern SPECT devices is between 0.35 and 0.7 mm (Aoi et al., 2006; Khalil et al., 2011; Chen et al., 2017). Moreover, SPECT isotopes have a longer half-life, chemical stability and the emission of photons with specific energies determined by the isotope in use. Thus, SPECT radiopharmaceuticals are easily transportable as long as they are not synthesized on site. In addition, they allow longer data acquisition periods and the use of multiple tracers which, in the case of PET, was not possible because of the fixed photon energy of annihilation (Cunha et al., 2014). The latter, however, are less expensive than the former ones in terms of development and radionuclide costs.

In general, an additional aspect to consider is that the amount of chemical compound applicable per body mass is limited; excessive amounts of radionuclides might interfere with the physiological dynamic state of body constituents (e.g., by occupying more than 5% of the total receptor sites) and threatening the validity of the tracer principle (Henriksen and Drzezga, 2011). Furthermore, the maximal injected volume should not exceed 10% of the total blood volume in order to obviate adverse effects on circulation (Hume et al., 1998).

## Neuroimaging of Brain Receptors

Several neuroimaging techniques are used in neuroscience research, from functional magnetic resonance imaging to systems like SPECT and PET. Small laboratory animals, especially rodents, are commonly used in brain research: they provide excellent models of human brain disorders and are essential to test potential therapeutic strategies. In this context, the development of high-resolution SPECT and PET systems for small-animal imaging provides important information, as for instance these techniques allow to study brain function and activity during the performance of behavioral tasks. Particularly, SPECT is an excellent tool to understand the pathophysiological bases of brain disorders. Moreover, as mentioned earlier, SPECT over PET has the ability to reach a spatial resolution below 1 mm, thus enabling detailed studies of the structure and function of different brain regions in animal models. Additionally, due to the relative longer half-lives of its radioligands, it is possible to assess prolonged dynamic function studies also by providing a simultaneous dual tracer imaging. Furthermore, the use of pinhole SPECT enhances the spatial resolution and, consequently, improves the image quality. Specific SPECT radioligands have been used to study different neurotransmitter systems (Lever, 2007; Khalil et al., 2011; Mathe et al., 2013). As SPECT, PET performs non-invasive, *in vivo* measurements of various molecular brain processes. This functional neuroimaging method has broken new ground in order to study brain physiology and pathology, ranging from research in animal models to clinical studies in humans. Moreover, by using the available radioisotopes, is it possible to perform biochemical and physiological measurements such as blood flow perfusion, glucose metabolism, density and affinity of presynaptic and postsynaptic receptors, neurotransmitter release, enzyme activity, drug delivery and uptake, and gene expression.

In Table 3, the most commonly used radiotracers in neuroimaging of brain receptors are listed. In addition, PET represents the most selective and sensitive method to measure molecular interactions due to its picomolar and nanomolar range (Petersen et al., 1988; Rinne et al., 2000; Acton and Kung, 2003; Rubins et al., 2003; Schaffhauser et al., 2003; Reeves et al., 2005; Schiffer et al., 2007; Saijo et al., 2009; Saigal et al., 2013). Because of its high resolution and molecular selectivity, small-animal PET brain imaging is optimal for detailed brain local analysis in rodents also allowing repetitive follow-up.

**Table 3:** Several brain radiotracers currently used in small animal neurological imaging (adapted from Brooks, 2005).

	**Radiotracer**	**Target/measured process**
SPECT	^99m^Tc-ECD ^99m^Tc-HMPAO [^123^I]IMP	Regional cerebral perfusion
	^111^In-Pentetate	Cerebrospinal fluid kinetics
	^99m^Tc-HYNIC – annexin V	Phosphatidylserine – dementia
	[^123^I]IBZM [^123^I]epidepride	Dopamine D2, D3 receptors
	[^123^I]ioflupane [^123^I]altropane ^99*m*^Tc-TRODAT	Dopamine reuptake transporter
	^123^I-β-CIT ^123^I-FP-CIT (DaTscan)	Dopamine transporter (DAT)
	[^123^I]AM281	CB1 cannabinoid receptors
	[^123^I]PK11195	Peripheral benzodiazepine receptor (PBR)
	[^123^I]IMPY	Amyloid
	[^123^I]IDAM [^123^I]ADAM	Serotonin teuptake transporter (SERT)
	[^123^I]iomazenil	GABA receptor
PET	[^18^F]FDG	Glucose metabolism
	[^15^O]H_2_O	Cerebral blood flow
	[^18^F]FluoroDOPA	Dopamine metabolism
	[^11^C]SCH 23390	D1 receptor
	[^18^F]Fallypride	D2 receptor
	[^11^C]Raclopride	D2/3 receptors
	[^11^C]PE2I	Dopamine transporter
	[^11^C]DASB	Serotonin transporter
	[^11^C]Carfentenil [^18^F]Cyclofoxy	Opioid μ sites
	[^11^C]Diprenorphine	Opioid μ, κ, and δ sites
	[^11^C]NMPYB	Muscarinic acetylcholine receptor
	[^11^C]Flumazenil	GABA_A_/benzodiazepine receptors
	[^11^C]PIB	b-amyloid deposits

One of the most interesting applications of preclinical PET and SPECT functional modalities is represented by the *in vivo* analysis of brain activity while the animal is performing a given behavioral task. Indeed, in this case radiotracers are used for the assessment of local changes in the release of neurotransmitters associated with the performance of the behavioral task. The study of the changes in metabolic activity and in neurotransmitter expression in various brain areas during task-induced neural activation is a key factor for understanding brain function in health and disease states and for exploring new potential drugs for brain disorders (Sharma and Ebadi, 2008).

In this section, we will give a brief overview of the neuroimaging techniques applied to the study of different neurotransmitter systems. As an example, we focus on opioid, dopaminergic and cannabinoid neurotransmission because these neurotransmitter systems, which are often disrupted in brain diseases, have been widely investigated through rodent imaging studies.

### Neuroimaging of Opioid Receptors

Opioid receptors (ORs) are widely distributed in the CNS and peripheral sensory and autonomic nerves. Their activation mediates a variety of functions such as pain reactivity, stress responses, natural and drug reward, learning and memory, and sociability; accordingly, ORs play an important role in several brain disorders (Van Ree et al., 1999; Law et al., 2000; Williams et al., 2001; Henriksen and Willoch, 2008). Preclinical neuroimaging studies related to the endogenous opioid system have been mainly performed in rodents. An example of application of rodents' brain imaging related to the endogenous opioid system lies in the multitudes of studies performed on pain processing and modulation. Pain is a complex experience encompassing sensory, affective and cognitive elements that are thought to be mostly regulated by μ-ORs in cortical and subcortical brain regions. fMRI and PET have both been used in rodent studies to shed light on different aspects of pain perception and modulation. These types of studies emerged to be somehow back translational, as an extensive literature on human brain imaging and pain exists, but still, rodents imaging proved to be essential to investigate the clinical phenomena in controlled situations. So far, imaging studies focused on pain activation patterns, pharmacological and non-pharmacological treatment of pain, and studies of morphological changes related to chronic pain have been performed in rodents providing invaluable information (Thompson and Bushnell, 2012).

In this context, fMRI BOLD was used to evaluate the effects of opiates such as morphine, proving that it was able to reduce pain-evoked brain activation following different painful stimulations (i.e., formalin, Tuor et al., 2000; capsaicin, Shah et al., 2005; electrical stimulation, Chang and Shyu, 2001) in rodents. Moreover, by fMRI studies both morphine and nociceptive stimuli were found to activate brain areas such as periaqueductal gray (PAG), amygdala and thalamus, which are brain areas directly involved in pain (Shah et al., 2005). Examples of the use of PET imaging in pain research came from studies aimed at measure cerebral metabolic changes related to nociception *in vivo*, using rats (Shih et al., 2008). Moreover, measuring glucose metabolism and ORs binding levels with PET imaging allowed to analyze neuropathic pain in the rat whole brain (Kim et al., 2016; Thompson et al., 2018).

Although small animal neuroimaging techniques studied the endogenous opioid system mainly in the context of pain research, they have been applied to other neuroscience fields as well. For instance, different drug addiction studies in rodents used PET (Itzhak, 1993; Melichar et al., 2005; Hooker et al., 2009; Placzek et al., 2015; Auvity et al., 2017, 2019) and fMRI (Cosa et al., 2017; Iriah et al., 2019) to investigate some aspects underlying addiction. Just to mention few examples, PET neuroimaging was used to investigate the brain metabolic changes following chronic morphine administration, to image ORs occupancy by methadone or to investigate the regulation of brain ORs by non-opioid drugs such as cocaine (Itzhak, 1993; Melichar et al., 2005; Chen et al., 2018). Moreover, PET techniques in rodents proved to be useful to study the effects of specific selective OR agonists such as the potent and highly selective κ-OR agonist salvinorin A (Hooker et al., 2009; Placzek et al., 2015). Interestingly, by PET imaging the hypothesis that a correlation between the immune system and morphine exposure and withdrawal exist was studied (Auvity et al., 2017, 2019). In this context, using translocator protein 18 kDa (TSPO) radioligand (a microglial activation detector) for PET imaging, Auvity and colleagues studied the neuroimmune component of opioid addiction revealing that there is not a detectable microglia activation during morphine tolerance and withdrawal (Auvity et al., 2017, 2019). As for fMRI, a study performed in rats revealed differences in brain when oxycodone was presented to either drug naïve rats or rats with prior, repeated exposure to the drug (Iriah et al., 2019). Similarly, fMRI was used to study the involvement of the endogenous opioid system in a rat model of heavy alcohol drinking (Cosa et al., 2017).

**Figure 4:**
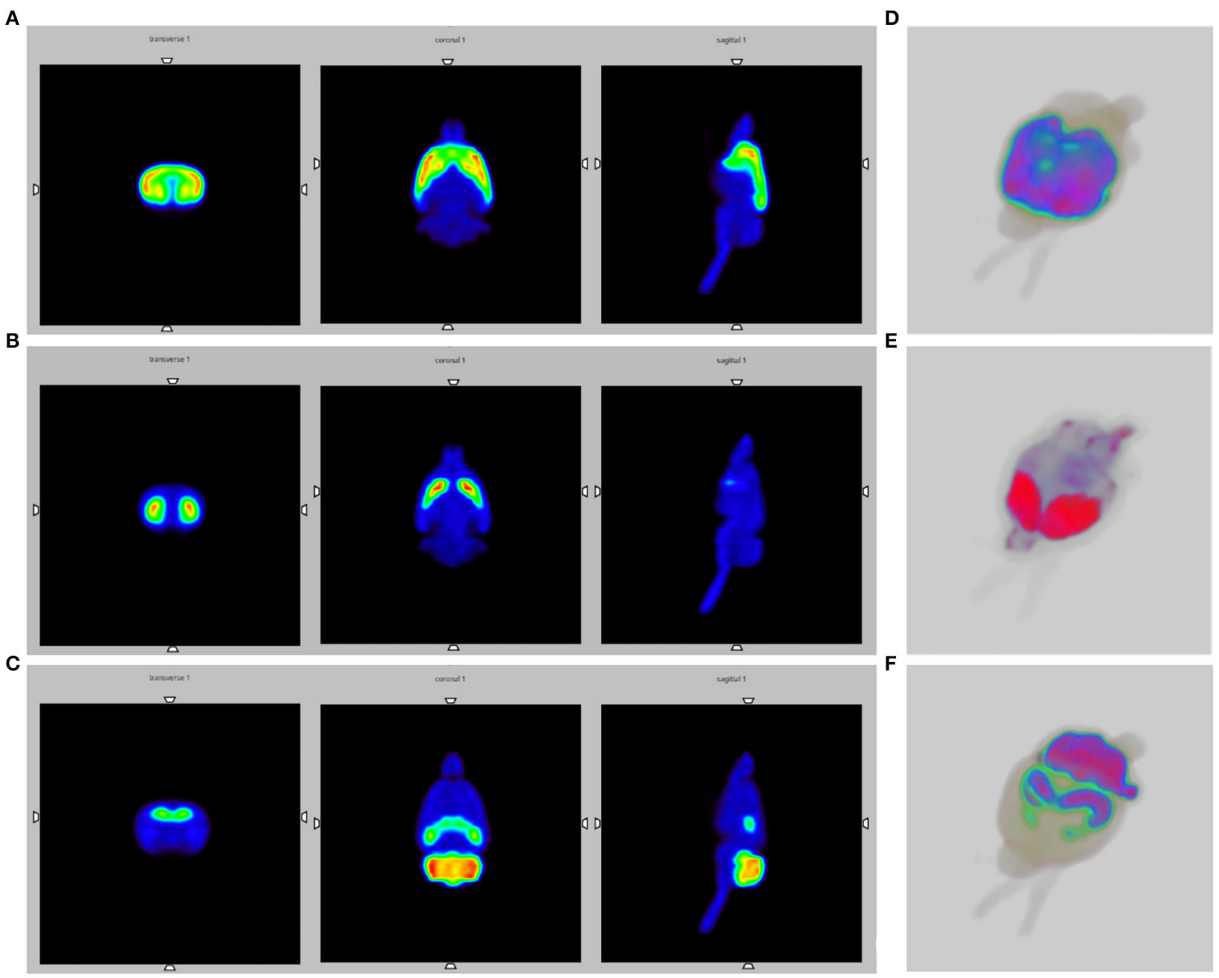
SPECT brain images obtained by Monte Carlo simulations, using the WHS SD Rat Brain V 2.0 phantom. **(A)** Slice images of the activated striatum and cerebral cortex for the opioid system; **(B)** Slice images of the activated striatum for the dopaminergic system; **(C)** Slice images of the activated thalamus, cerebellum and globus pallidus for the endocannabinoid system; **(D–F)** 3D SPECT reconstructions.

Figure 4A shows the Monte Carlo simulation slice images of the activated striatum and cerebral cortex for the endogenous opioid system.

### Neuroimaging of Dopamine Receptors

The dopaminergic system plays a pivotal role in several brain functions, such as motor control, motivation and reward processes, cognitive function and reproductive behaviors (Schultz, 2007). Since so many functions are dependent on dopaminergic neurotransmission, it is not surprising that alterations of dopaminergic signaling have been involved in many if not all neurological and psychiatric disorders (Beaulieu and Gainetdinov, 2011; Klein et al., 2019). Therefore, brain imaging studies in rodents combined with the use of radioligands targeting both presynaptic and postsynaptic dopaminergic markers have been extensively performed to investigate the dopaminergic synapse *in vivo*. For instance, studying dopaminergic neurotransmission by imaging techniques in laboratory animals provided invaluable information on the control of nigrostriatal and mesolimbic dopaminergic activity by other neurotransmitter systems, such as glutamate and GABA (Nikolaus et al., 2017, 2018, 2019).

As an example, we will now briefly discuss how imaging studies of dopaminergic neurotransmission contributed to addiction research.

Neuroimaging studies in rodents offer the possibility to assess brain activation in response to drug administration in both naïve and drug-exposed subjects providing deeper insight into the mechanisms underlying addiction. Thus, neuroimaging techniques can assess acute and long-term effects of drugs of abuse (Michaelides et al., 2012). fMRI experiments provided substantial understanding of the brain response to drugs of abuse in naïve or drug-exposed subjects, even if the requirement of motionless scanning limited the studies to the pharmacological effects of the drugs in the anesthetized state (Michaelides et al., 2012). Similarly, PET studies performed in rodents largely contributed to the understanding of the involvement of the dopaminergic system in drug addiction. For instance, some of the firsts studies performed with PET imaging in rodents showed changes in D1 and D2 dopamine receptor availability in response to chronic cocaine exposure (Tsukada et al., 1996) and cocaine withdrawal (Maggos et al., 1998). Moreover, PET imaging experiments assessed the relationship between changes in brain dopamine levels and the preference for a conditioned contextual stimulus associated with cocaine administration in rats (Schiffer et al., 2009). Thus, rodents exposed to drug cues presented increased dopamine levels in the ventral striatum, similarly to humans, in which discrete drug cues associated with cocaine elicited increased striatal dopamine levels (Volkow et al., 2010; Michaelides et al., 2012). Besides fMRI and PET imaging, preclinical SPECT studies also contributed to drug addiction research. For instance, using [^123^I] IBZM single pinhole SPECT, the imaging of the D2 dopamine receptor availability and endogenous dopamine release was proven feasible in mice (Jongen et al., 2008).

Rodent studies have been performed to test the validity of novel tracers for the dopamine transporter (DAT) but also to provide information about addiction mechanisms. For example, PET imaging in mice was used to study DAT occupancy after intravenous doses of cocaine and methylphenidate (Gatley et al., 1999), or to disclose the effect of acute cocaine administration on pre-synaptic dopamine function (Bonsall et al., 2017). Moreover, imaging DAT with PET was useful to reveal the involvement of dopamine signaling in the effects of chronic methamphetamine on psychomotor and cognitive functions in rats (Thanos et al., 2017). As well as PET imaging, SPECT imaging on DAT helped to shed light on the brain mechanisms underlying drug addiction. Thanks to SPECT studies, Nikolaus et al. estimated the reuptake and the release of DA in the rat striatum by assessing the competition between endogenous DA and administered DAT and D2 receptor radiotracer (Nikolaus et al., 2016). Similarly, SPECT imaging was used to investigate DAT binding characteristics after treatment with methylphenidate in rats (Nikolaus et al., 2005a, 2007). Imaging studies on DAT proven to be useful also in the study of new generation psychostimulants. Indeed, DAT fMRI imaging was used to assess functional connectivity and dopaminergic alterations in the rat brain after exposure to new generation psychostimulant drugs such as methylenedioxypyrovalerone (MDPV), a synthetic cathinone which is known to act as a potent uptake inhibitor at plasma membrane transporters for dopamine (Colon-Perez et al., 2016, 2018).

Figure 4B shows the Monte Carlo simulations slice images of the activated striatum for the dopaminergic system.

### Neuroimaging of Cannabinoid Receptors

The endocannabinoid system (ECS) is composed by the G-protein coupled cannabinoid receptors CB1 and CB2, the endocannabinoids, which are retrograde lipid messengers that move backward from postsynaptic to presynaptic sites, and the enzymes involved in endocannabinoid synthesis and degradation (Piomelli, 2003). Endocannabinoids regulate a plethora of brain function, from memory processes to emotional states, pain perception, motor activity, sociability, food intake and appetite, sleep and reward processes (Zou and Kumar, 2018). Moreover, the ECS has been found to have a role in several brain disorders including anxiety, depression, schizophrenia, multiple sclerosis, neurodegeneration, epilepsy, addiction, and autism spectrum disorder (Zou et al., 2019).

Although research on ECS imaging is still at early stages, fMRI, PET, and SPECT studies in the brain of humans and animals have been performed to investigate ECS activity in different contexts. These studies provided important information on biodistribution of cannabinoid drugs, cannabinoid receptor localization, cannabinoid drugs competition, ECS activity correlated to brain metabolism and clarified the impact of cannabinoid drugs on different neurotransmitter systems (Lindsey et al., 2005; Cilia, 2018).

Originally, *in vivo* PET or SPECT imaging of CB1 and CB2 cannabinoid receptors has been largely unsuccessful owing to the lack of high-affinity and high-selective radioligands (Van Laere, 2007). Most efforts in developing SPECT ligands for *in vivo* imaging of cannabinoid receptors were directed on radiotracers based on the structure of rimonabant (SR141716A), a CB1 receptor antagonist/inverse agonist, such as [^123^I]AM251and [^123^I]AM281 (Van Laere, 2007). Of these, [^123^I]AM281 was used to image CB1 brain receptors for the first time in primates and humans (Gatley et al., 1998; Berding et al., 2004). Recently, PET tracers such as [^11^C] OMAR (Wong et al., 2010), [^18^F] MK-9470 (Burns et al., 2007; Van Laere et al., 2009, 2010; Sanabria-Bohorquez et al., 2010), [^11^C] MePPEP (Terry et al., 2009), [^18^F] FMPEP-d2 (Terry et al., 2010) and [^11^C] SD5024 (Tsujikawa et al., 2014) have been used to image the CB1 receptors in humans and these ligands appear to have improved imaging properties (Cilia, 2018).

Imaging studies in rodents were aimed at evaluating the role of the ECS in different psychiatric and neurological disorders. For instance, the PET CB1 receptor ligand [^18^F]-MK-9470 was used in rats to investigate the role of the ECS in epilepsy (Goffin et al., 2008), to assess changes in its binding to CB1 receptors in the brain of the 6-hydroxydopamine rat model of Parkinson's disease (Casteels et al., 2010), in a rat model of anorexia nervosa (Casteels et al., 2014), and to image CB1 receptors in a transgenic rat model of the early phases of Huntington disease (Casteels et al., 2011). Conversely, radiotracers such as the [^18^F]FMPEP-d2 were used to investigate age- and genotype-dependent impairments in the availability of CB1 receptors in a mouse model of Alzheimer's disease (Takkinen et al., 2018).

CB2 expression in microglia is known to significantly increase during neuroinflammatory conditions (Savonenko et al., 2015; Navarro et al., 2016). As a consequence, several tracers have been recently examined in animal models of microglial activation. For instance, [^11^C]A-836339 (Yao et al., 2009; Horti et al., 2010) was used to study CB2 receptor activation in mice and rats treated with lipopolysaccharide (LPS), a bacterial compound known to activate the immune system (Horti et al., 2010; Pottier et al., 2017). More recently, an ^18^F-labeled analog of A-836339, [^18^F]29 was also used to image CB2 receptors in the brains of mice treated with LPS, but its further application in imaging studies was limited by its fast-metabolic degradation (Moldovan et al., 2016). However, an increased tracer uptake after systemic administration of LPS with both [^11^C]RS-016 and [^11^C]RSR-056 was found in the mouse brain (Slavik et al., 2015, 2016).

Alternative targets to study the ECS by neuroimaging techniques are substrates for enzymes involved in endocannabinoid degradation, transport or synthesis. Two examples are the MAGL ligand [^11^C]10 (MAGL-0519), which demonstrated high specific binding and selectivity *in vitro* and *in vivo* (Cheng et al., 2018) and the radiolabeled reversible FAAH inhibitor [^11^C]MPPO, which showed moderate uptake with heterogeneous distribution in rodents brain (Wang et al., 2016).

Figure 4C depicts the Monte Carlo simulation slice images of the activated thalamus, cerebellum and globus pallidus for the ECS. Moreover, Figures 4D–F provide 3D SPECT reconstructions, respectively of the opioid, dopaminergic and endocannabinoid system.

## Neuroimaging in Laboratory Animals to Shed Light on Brain Disorders

Brain imaging in small laboratory animals represents one of the most complex scenarios in SPECT/PET techniques due to the small details of brain structures, relatively low tracer uptake and complex kinetics. Brain imaging requires two main conditions, namely the highest possible spatial resolution and sensitivity of the measurement instrument. The effectiveness of these conditions strictly depends on the size of the brain; indeed, a small brain implies a limited FOV, thus optimizing resolution and sensitivity. In order to obtain a proper neuroimaging, several factors must be taken into consideration, in the light of the poor brain radiotracer uptake (about 1%), such as dynamic imaging with good temporal resolution and reasonable counting statistics.

In this context, the first PET tomographs designed for small animal imaging applications were the Hamamatsu SHR 2000, developed by Hamamatsu (Hamamatsu, Japan) in the 1990s, as well as the ECAT-713, developed by CTI PET Systems Inc. (Knoxville, TN). Both systems were characterized by a large radial FOV provided by large detector rings. For this reason, they were preferably used with non-human primates. The real ground-breaking technologies were represented by the microPET (Nandi et al., 2017) and RatPET (Woody et al., 2007a,b; Schulz and Vaska, 2011) systems, which both significantly contributed to the understanding of brain disorders such as Alzheimer's disease (Zimmer et al., 2014), Parkinson's disease (Opackajuffry and Brooks, 1995; Opackajuffry et al., 1996; Sullivan et al., 1997; Nguyen et al., 2000; Bjorklund et al., 2002; Inaji et al., 2005) and Huntington's disease (Araujo et al., 2000; Ishiwata et al., 2002a,b).

**Figure 5:**
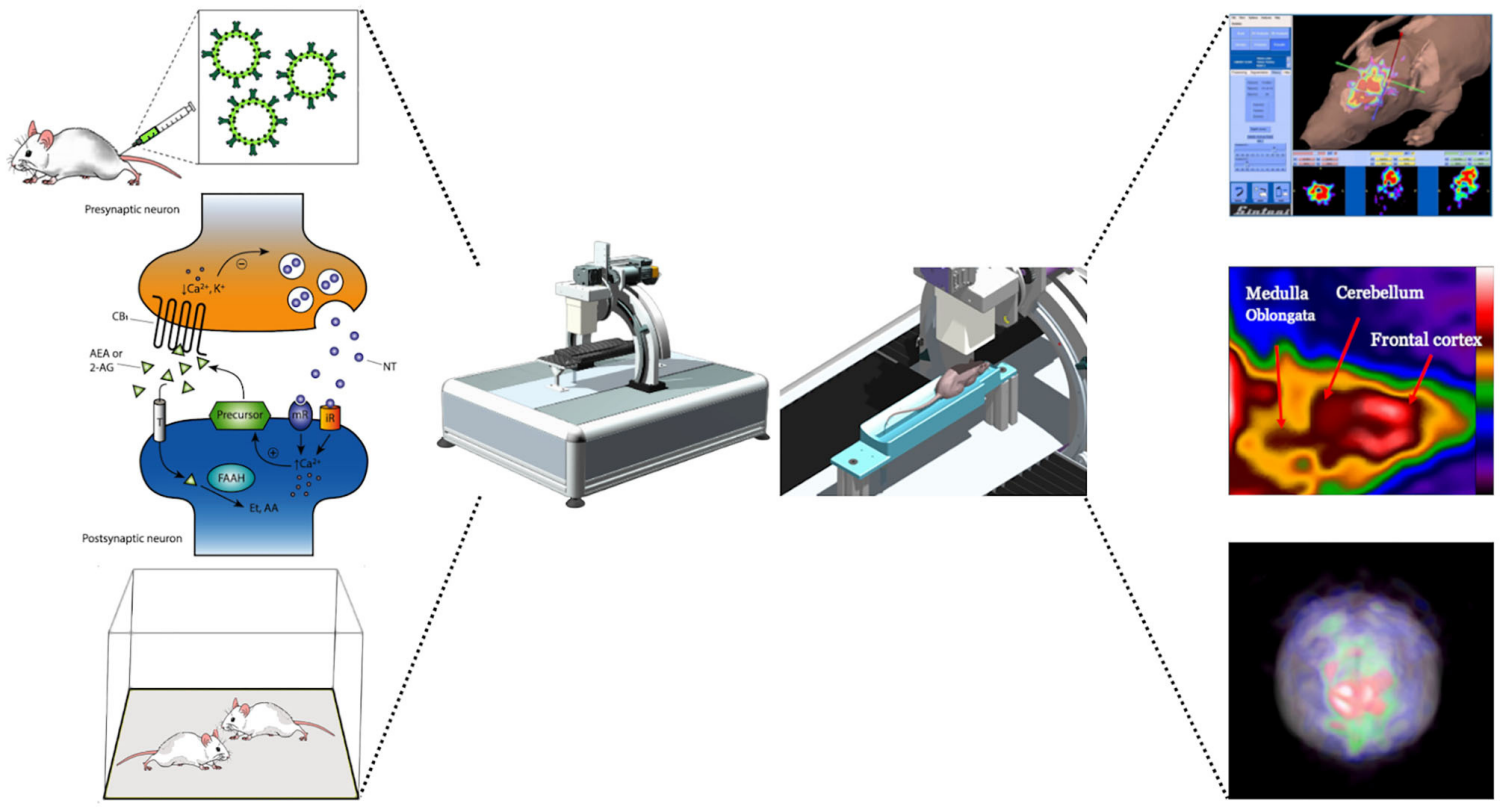
Schematic illustration of a potential experiment to map neuronal activity by using a SPECT system. After the radiotracer is injected via the tail vein, the experimental subject undergoes behavioral testing and the neuronal response can be obtained from changes in receptor occupancy levels using SPECT imaging. Images shows changes in receptors signal following the behavioral responses.

Pinhole SPECT systems perform accurate and quantitative imaging results from mice (Acton et al., 2002a,b) and rats (Booij et al., 2002). Furthermore, the multimodality represented by the coupling of SPECT/MRI provides valuable results for the study of brain substructures (Booij et al., 2003; Scherfler and Decristoforo, 2006; Nikolaus et al., 2018). The use of increasingly more innovative devices is required to rapidly acquire the radiotracer dynamic for capturing the true shape of the time–activity curve, associated with high spatial resolution in order to visualize a proper separation between neighboring brain regions. Figure 5 summarizes the process of mapping neuronal activity by using a SPECT system.

In this context, multipin-hole multiple-detector stationary SPECT systems have been developed in order to overcome the limits of traditional imaging (Furenlid et al., 2004; Vastenhouw and Beekman, 2007). Despite limited evidence on the effect of radiation dose on laboratory animals, brain imaging shows significant applicability in multi-pinhole SPECT systems due to the higher doses required for sufficient counting statistics during dynamic or quantitative SPECT acquisition. To this purpose, several simulations have been carried out in order to reach the optimal detector configuration for mouse brain imaging (Franc et al., 2008).

### Limitations of Neuroimaging in Anesthetised Animals

Imaging studies often require the partial or total immobilization of the experimental animal. Besides rising serious ethical concern, the effects of restrain in rodents have been found to cause a considerable distress, even if animals are trained and habituated several days before the acquisitions to the scanner environment. In rodents, restraint increases both corticosterone levels and the activation of the central nucleus of the amygdala, which regulates pain sensation and influence behavioral responses (Low et al., 2016). Thus, training animals to the scanner environment with short-term repeated restrain periods in attempt to reduce distress during the scanning procedure may cause long-lasting neurochemical and neuroendocrine changes, potentially modifying the physiological responses seen in awake rodents compared to anesthetized animals (Tremoleda et al., 2012; Low et al., 2016).

For this reason, the use of anesthesia to perform imaging experiments has been introduced, as it is able to remove movement artifacts and to reduce stress effects (Pawela et al., 2008; Sommers et al., 2009; Grandjean et al., 2014). Small animal imaging techniques commonly use two kinds of anesthetics: injectable and inhaled. The latter, most often Isoflurane and Halothane, show fast onset and recovery times, precise control of doses and maintenance times of anesthesia, and they are quickly eliminated. Instead, the former such as Fentanyl and Ketamine are more suitable for long-term imaging such as SPECT/PET acquisitions. Their dose depends on several variables (e.g., age, sex, strain, experimental set-up etc.), and they need continuous monitoring for dose adjustments (for a review see Tremoleda et al., 2012). However, it must be taken into account that anesthesia might cause several effects that alter the experimental outcomes, especially in behavioral studies, in which it is fundamental to investigate emotional alterations, stress, pain and any behavioral parameter arising from experimental tasks. As an example, anesthesia depresses cortical activity and affects regional cerebral blood flow and metabolism rate of oxygen and glucose, thus affecting the experimental results (Lahti et al., 1999; Nakao et al., 2001; Sicard et al., 2003; Masamoto et al., 2007). Furthermore, general anesthetic drugs produce extensive neuronal changes by affecting both inhibitory and excitatory neurotransmission (Pocock and Richards, 1993; Campagna et al., 2003), and they cause neuronal apoptosis as well as changes in dendritic morphology in the developing brain of rodents causing long lasting brain alterations (Jevtovic-Todorovic et al., 2003; Wise-Faberowski et al., 2005; Yon et al., 2005; Briner et al., 2010; Liang et al., 2010). For these reasons, the brain alterations and the changes in neurotransmission induced by anesthesia could hinder data interpretation. In line with this possibility, various PET studies have shown that anesthetic agents modify the binding of dopamine receptor radioligands (Hassoun et al., 2003; Momosaki et al., 2004). In order to bypass these limitations, a novel protocol has been developed based on the free movement of rodents preinjected and scanned under light anesthesia (Schiffer et al., 2009). In order to allow proper radiotracer distribution and uptake, animals have to be in state of consciousness, therefore anesthesia can be injected immediately after the radiotracer uptake and before the beginning of the scanning session, to avoid the confounding effects of anesthetic drugs.

Another vulnerable aspect of the imaging systems is linked to the resolution itself, which can be subjected to interferences caused even by imperceptible movements. An additional parameter that must be taken into consideration is represented by the region of interest (ROI) which corresponds to a distinct structure within the brain. It cannot be underestimated that, even if successive acquisition is done, the position and geometry of the identified ROI may change because of differences in radiotracer uptake, statistical noise, blood flow and influence of surrounding brain regions affecting the voxel intensities in the ROIs. In the light of these factors it is not possible to give a univocal definition of ROI due to its irreproducible results, particularly if working with ROIs close in size to the resolution of the scanner (Pascau et al., 2009). This problem can be overcome by aligning PET images of the same animal such that one set of ROIs may be used for all image sets of that animal. Ideally, this could be achieved also by placing the rat brain in exactly the same position within the scanner for each scan. Therefore, plastic stereotactic frames have been designed for reproducible positioning in unconscious rodents imaging enabling accuracy in identifying anatomical structures from a reference template and a rodent brain atlas, as well as in repositioning the same animal in longitudinal studies (Ponsky et al., 2002; Lancelot and Zimmer, 2010).

## Nuclear Imaging on Conscious Animals

As discussed in the previous section, the use of anesthesia before tracer injection poses some limitations.

More recently, two alternative approaches have been proposed in order to minimize animal movement during imaging and improve the quality of the experimental results (Schulz et al., 2011).

**Figure 6:**
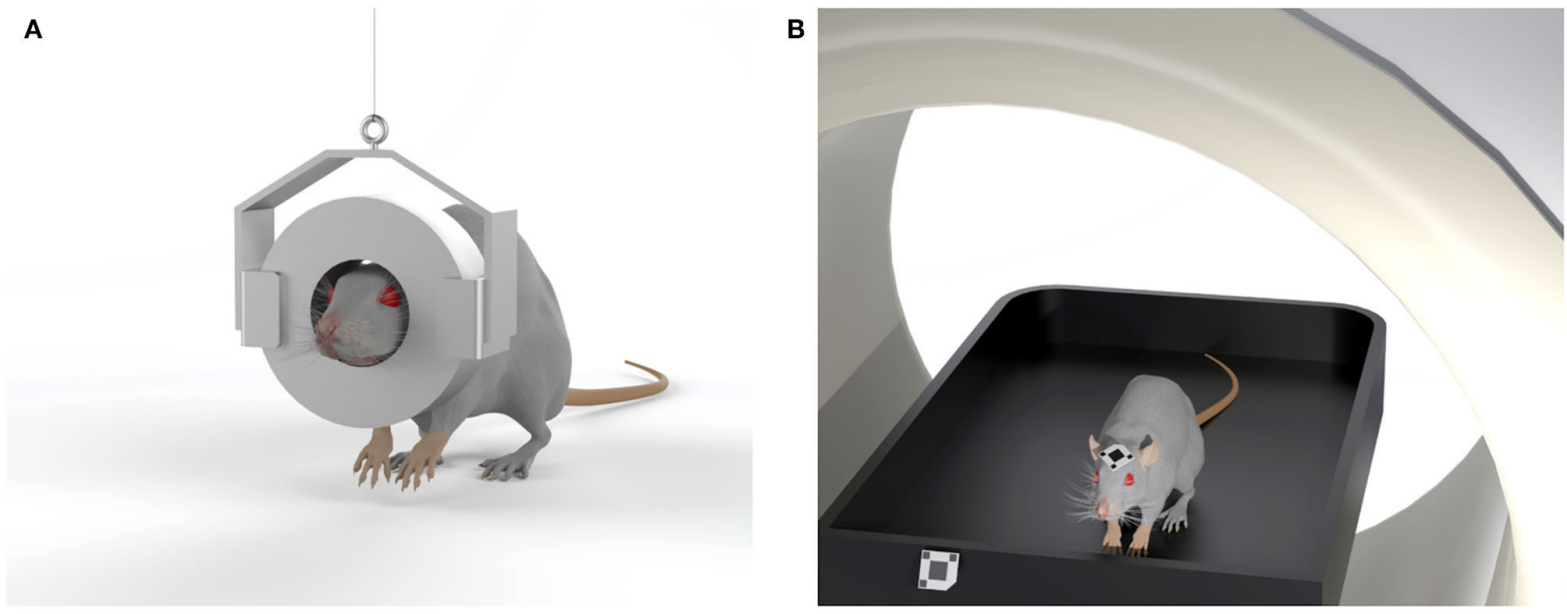
Sketches of two systems designed to eliminate the need for anesthesia in rodent studies. **(A)** The RatCAP tomograph (rat conscious animal PET) consisting of a miniaturized full-ring PET scanner which is attached directly to the head, covering nearly the entire brain; **(B)** The open-field PET system, based on a commercial preclinical scanner, enables brain scans of rodents in motion due to an optical motion tracking and a custom designed motion-adaptive animal enclosure attached to a robotic arm.

The first proposed device, allowing imaging experiments to be performed on rodents without the use of anesthesia, consisted of a small, head-mounted tomograph which allowed PET imaging of the brain of an awake animal. This “wearable” PET system, known as the RatCAP, was developed by a team of researchers led by the physicist Paul Vaska and allows scans on awake, freely moving rats (Vaska et al., 2004; Woody et al., 2004; Kyme et al., 2008). Briefly, the tomograph consists of a 4 cm diameter engineered PET ring directly mounted on the rat's head, with a weight of about 250 g. The detector ring and the readout electronics mounted to the head of the rat are supported by a tether that carries the weight and provides a pathway for electrical signals. An additional mechanical mobility system supports the overall weight of the scanner and allows the rat to move freely around a 40 × 40 cm behavioral chamber while PET images are acquired. Even if the rat can move in the observational chamber, there are still some limitations in terms of where the animal can go and concerning the shape and weight of the device that might undermine its use for certain experiments, such as those that involve nose-poking into narrow spaces in search of food rewards. The first practical application of the RatCAP was to observe changes in dopamine levels during behavioral tasks (Schulz and Vaska, 2011), but further improvements are needed to lightweight the system and allow more degrees of freedom to the animals, in order to apply this technology to a broad range of behavioral studies using radiotracers to track different neurotransmitters (Figure 6A).

The second proposed technology consists of a small animal PET detector system that surrounds a chamber coupled with a tracking system that allows the position of the rodent's head within the chamber to be measured over time (Schulz et al., 2011; Kyme et al., 2019; Herfert et al., 2020). This system, known as Open-field PET, combines motion-compensated PET with a robotically-controlled animal enclosure to enable simultaneous brain imaging and behavioral recordings in unrestrained rodents. In detail, the animal is free to walk around the chamber during an imaging session. The open-field PET system is made up of a commercial PET scanner, an optical motion tracking and a custom designed motion-adaptive animal enclosure attached to a 6-axis robotic arm (Kyme et al., 2019). Since a small lightweight marker is attached to the forehead of the animal, the motion tracking is able to measure the changing position and orientation of the animal inside the chamber. For image reconstruction, the event-by-event tracking information is used to align the data detected by the PET scanner through the use of a reconstruction algorithm to form a coherent animal body volume (Yao et al., 2012; Herfert et al., 2020). This technique, therefore, allows simultaneous assessment of changes in brain function and behavioral responses to external stimuli in awake, unrestrained animals, with broad applications to behavioral neuroscience research (Figure 6B). Indeed, the two techniques described here pave the way to a wide range of experiments to correlate brain function and behavior in response to a wide variety of interventions in freely moving, non-anesthetized rodents.

## Challenges and Perspectives

Imaging in laboratory animals represents a valuable tool in neuroscience research. Indeed, a non-invasive, *in vivo*, qualitative and quantitative analysis allows to perform innovative studies to shed light on brain function in health and disease states.

Dopaminergic neurotransmission has been widely investigated through small animal neuroimaging due to the large availability of tracers targeting plasma membrane transporters and receptor binding sites. Furthermore, we also provided evidence that other neurotransmitter systems, such as the endogenous opioid and the ECS, have been studies through imaging technologies in rodents.

SPECT and PET technologies provide a dynamic assessment of radiotracer biodistribution into brain substrates and tracer uptake can provide important information in terms of availability of the targeted molecular entity in relation to a given physiological or pathological process. Particularly, SPECT imaging offers several advantages: its characteristics of high spatial resolution, reduced investigation times as well as high detection efficiency and radiotracers availability contribute to its wide application in preclinical research. Nevertheless, PET techniques play a key role in the study of brain activity and plasticity in rodents, since they allow to correlate behavioral outputs with changes in neurotransmitter activity during the execution of a defined task. For these reasons, PET imaging technology can provide a significant contribution to neuroscience research, despite it also presents limitations such as the lack of enough spatial resolution to enable a more detailed study of changes in regional activity within certain brain structures and the short half-life of its radiotracers.

The integration of morphological and functional techniques, such as SPECT, PET, CT, and MRI, will allow to overcome the limitations that characterize each of these individual techniques, by correlating the functional characterization of brain disorders at the circuit level and providing more detailed information about smaller structures in the animal brain. Furthermore, longitudinal *in vivo* investigation carried out using these technologies drastically reduces the number animals involved in the experiments. Another important aspect to consider is the influence of anesthesia on preclinical imaging studies. Indeed, the anesthetic drug can modify several parameters, such as the cerebral blood flow or receptor binding and, hence, affect the SPECT/PET results. For this reason, in order to reduce the influence of anesthesia, innovative animal PET scanners in freely moving animals are being developed. Above all the proposed technologies for the simultaneous assessment of dynamic brain processes and behavior, RatCAP and the Open-field PET represents the two most successful examples. Both systems allow the whole brain to be imaged while the animal is awake and involved in a given behavioral task. Although the resolution of these systems is sufficiently sensitive for brain imaging, further studies are needed for the real-time detection of changes in receptor occupancy related to behavioral outputs.

In the near future, we believe that two types of enhancements might be possible: the first regards the improvement of the standard systems resolution, the second concerns the development of more specific detectors applied just on certain brain areas in order to better study a particular receptor activation. Specifically, the necessary improvements concern the development of lighter devices that can be worn by the animal without compromising the degrees of freedom necessary for carrying out a behavioral task and the development of *ad-hoc* detectors to provide information on specific brain areas, also considering the difficulty of an imaging synchronized with the motion tracking.

Therefore, it is necessary to improve the spatial resolution of the current imaging systems and to further reduce the weight of new generation detectors. The solution we propose to improve resolution is the application of new methods such as the Super Spatial Resolution (SSR), that provide images with a greater content in terms of spatial resolution and consequently of diagnostic details which is essential for studying brain circuits involved in brain disorders (Trinci et al., 2010; Massari et al., 2018, 2019). Indeed, the use of the SSR method combined with the use of new optoelectronic components can lead to millimeter or sub-millimeter spatial resolutions in devices based on the parallel hole collimation, leading to the development of innovative detectors competitive with those based on pinhole or multipinhole collimators.

The introduction of new small photodetectors, such as silicon photomultipliers with size of 1 × 1 mm, allows to develop a novel class of detectors able to lighten the weight applied on the head of the animal and providing the imaging of only certain brain areas supposedly involved in specific brain functions. In our opinion, it is very likely that brain mapping will be carried out by means of innovative multiple detector systems. Once the brain area of interest will be identified by means of traditional imaging on the unconscious animal, single detectors will be applied on this area in order to further measure the count rate (the measured radioactive radiation dose actually reaching the sensor), while the conscious animal performs the behavioral tasks. In this way, it will be possible to identify the radioactivity uptake over time, thus obtaining a brain mapping of the circuits involved in a given activity.

Even though SPECT and PET alone do not guarantee the absolute quantification of receptor and neurotransmitter changes, their application combined with other *ex vivo* methods, including immunohistochemistry, HPLC and microdialysis, has significantly advanced neuroscience research. Moreover, the development of new radiopharmaceuticals and the adoption of novel animal models with optimized imaging techniques, have made SPECT and PET powerful investigation tools for the study of neurochemical mechanisms underlying brain disorders.

What is particularly ground-breaking in imaging technologies is their transnationality in both animals and humans, which makes it desirable for these techniques to be ultimately applied to behavioral neuroimaging in human beings.

## Author Contributions

AD'E: conceptualization. AD'E and SS writing-original draft preparation. AnS edited the draft manuscript. VT and RM methodology. VT, RM, and AlS supervision, writing-review, and editing. All authors contributed to the article and approved the submitted version.

## Conflict of Interest

The authors declare that the research was conducted in the absence of any commercial or financial relationships that could be construed as a potential conflict of interest.
